# Supplementary material for: The genome sequence of Slender Tare, Vicia parviflora Cav. (Fabales: Fabaceae)
Source: Wellcome Open Res. 2026 Sep 17;11:696. [Version 1] doi: 10.12688/wellcomeopenres.27839.1 (PMC13658993; doi:10.12688/wellcomeopenres.27839.1)
Supplement: Peer review history

# Reviewer report: Reviewer response for version 1

**DOI:** 10.21956/wellcomeopenres.30634.r170893

**Published:** 2026-10-12

**Authors:** Lian‐Ming Gao

**Affiliations:** 1 Kunming Institute of Botany, Kunming, Yunnan, China

The manuscript presented a genome assembly of *Vicia parviflora* as well as its mitochondrial and plastid genome with NGS, PacBio HiFi and Hi-C sequence data. The reference genome is part of the Darwin Tree of Life project, which will provide genomic resources for genomics and biological research. I have several concerns on the manuscript as bellow:

 1. As Vicia parviflora has been treated as Ervum gracile DC.and adopted by Stace (2019), why the species name was used as Vicia parviflora not Ervum gracile.

 2. The HiFi data used for the primary assembly were obtained from individual drVicParv1, whereas the Hi-C and RNA-seq data were obtained from another individual, drVicParv2, collected from the same location. Although the overall heterozygosity of the species is very low (only 0.05%), intraspecific structural variations such as inversions, translocations, CNVs, and large insertions/deletions may still exist between different individuals. When the Hi-C data from drVicParv2 are mapped back to the contigs of drVicParv1, true structural differences between individuals might generate abnormal interaction signals, which could be misidentified as assembly errors, leading to biases in scaffold anchoring and affecting the reliability of the seven chromosome pseudomolecules. The individual used for sequencing are different should be clarify in the manuscript.

 3. This study only generated transcriptome data from leaf tissue, not from tissues of flowers, stems and/or roots. Subsequent gene annotation may therefore lack transcript evidence from key tissues of flowers, stems and roots.

 4. I wonder why the Hifiasm still run with --primary but not with Hi-C integrated phasing using deep Hi-C data?

 5. The alternate haplotype assembly is only 40.44 Mb, indicating a very large difference from the primary assembly. Whether it means only including the residual haplotypes? Suggest renaming or provide complete phased assembly and its corresponding quality metrics.

 6. Manual curation reduced the scaffold N50 by 74.4%, which is a significant large decrease. Could the authors show the changes in assembly quality before and after curation?

Are the datasets clearly presented in a usable and accessible format, and the assembly and annotation available in an appropriate subject-specific repository?

Yes

Are sufficient details of the sequencing and extraction, software used, and materials provided to allow replication by others?

Yes

Are the rationale for sequencing the genome and the species significance clearly described?

Yes

Are the protocols appropriate and is the work technically sound?

Partly

Reviewer Expertise:

Plant Phylogeny and evolution, population genetics

I confirm that I have read this submission and believe that I have an appropriate level of expertise to confirm that it is of an acceptable scientific standard.

# Reviewer report: Reviewer response for version 1

**Published:** 2026-09-17

**Authors:** Automated Benchmarking

**Affiliations:** 1 Wellcome Sanger Institute, Hinxton, UK

As this article is a Genome Note, Automated Benchmarking has been used to determine whether the reported assembly passes four defined checks established by the Earth BioGenome Project: the raw and assembled data are publicly available, the Earth BioGenome Project three-part metric is met, the k-mer completeness exceeds 90%, and the BUSCO score passes the stated threshold. For Vicia parviflora (drVicParv1), all four checks passed. Data available: pass. ENA study PRJEB80417, raw-read accessions ERR13725956, ERR13725957, ERR13732198, and ERR13962545, and assembly accessions GCA_965231315.1 and GCA_965231235.1 are public. Earth BioGenome Project three-part metric: pass. The primary assembly is reported as 6.C.Q62, meeting the 6.C.Q40 reference standard. k-mer completeness: pass. The combined haplotype assemblies have 99.38% k-mer completeness, exceeding the Earth BioGenome Project target of 90%. BUSCO: pass. The primary assembly, which is proposed as the reference assembly for this species, has 97.6% complete BUSCOs (94.3% single-copy and 3.3% duplicated). BUSCO software version: 5.7.1; lineage dataset: fabales_odb10. It meets the plant-specific target of greater than 90% complete BUSCOs. This decision uses values reported in the Genome Note and public accession records.

Reviewer Expertise:

NA

I confirm that I have read this submission and believe that I have an appropriate level of expertise to confirm that it is of an acceptable scientific standard.
